# Applications of a Chronic Care Model for Self-Management of Type 2 Diabetes: A Qualitative Analysis

**DOI:** 10.3390/ijerph182010840

**Published:** 2021-10-15

**Authors:** Rashid M. Ansari, Mark F. Harris, Hassan Hosseinzadeh, Nicholas Zwar

**Affiliations:** 1 Faculty of Medicine, School of Population Health, University of New South Wales, Sydney, NSW 2052, Australia; 2 Centre for Primary Health Care and Equity, University of New South Wales, Sydney, NSW 2052, Australia; m.f.harris@unsw.edu.au; 3 Faculty of Medicine and Health, School of Health and Society, University of Wollongong, Sydney, NSW 2522, Australia; hassanh@uow.edu.au; 4 Faculty of Health Sciences and Medicine, Bond University, Gold Coast, QLD 4226, Australia; nzwar@bond.edu.au

**Keywords:** chronic care model, self-management of type 2 diabetes, chronic disease, healthcare system of Pakistan, patients’ quality of life, qualitative analysis

## Abstract

The main aim of this study was to explore the suitability, practicality, and acceptability of the self-management support and delivery system design components of the Chronic Care Model (CCM) in type 2 diabetes self-management in primary care settings in rural Pakistan. Thirty patients living with type 2 diabetes and 20 healthcare professionals (10 general practitioners and 10 nurses) were recruited from Al-Rehman Hospital at Abbottabad, Pakistan. The study data were collected using semi-structured interviews and analyzed using thematic analysis. The self-management element of the CCM played an important role in managing type 2 diabetes, and self-efficacy in relation to diet and diabetes management were the most effective strategies. Surprisingly, considering the local culture around diabetes, patient care reflecting their cultural background was identified as an important factor by patients not healthcare professionals. The delivery system design element of the CCM promoted multidisciplinary teamwork. Our findings suggest that the self-management support and delivery system design components of the CCM provided an effective framework for supporting diabetes self-management education and support in rural areas.

## 1. Introduction

According to the International Diabetes Federation, around 463 million individuals had diabetes mellitus in 2019, with a projected 700 million by 2040. Type 2 diabetes, the most prevalent type, accounts for approximately 90% of all diabetes occurrence worldwide. Globally, type 2 diabetes imposes a large economic burden, with expected yearly expenditures of USD 760 billion in 2019 and projected to climb by 11.2 percent to USD 845 billion by 2045 [1].

Type 2 diabetes mellitus is a serious health concern in Pakistan, which ranks among the top ten countries worldwide in terms of diabetes prevalence among adults aged 20 to 79 years [2]. The prevalence of lifestyle risk factors is 26.5% among males aged 50–59 years and 35% among females within the same age group [2,3]. The main modifiable behavioral risk factors are tobacco use, unhealthy diet, and physical inactivity [3,4].

The Chronic Care Model (CCM) was developed with the primary goal of improving the healthcare system and assisting individual and population health interventions. The model emphasizes patient-centered care and an interdisciplinary approach [5,6]. The model includes six key elements: community resources, delivery system design, health system, self-management support, clinical information systems, and decision support. However, only self-management support and delivery system design components were assessed in this study.

The review of the literature demonstrates that self-management support improves patient-level outcomes such as physiological indicators of disease, quality of life, health status, and satisfaction [7]. Patient self-management support (SMS) has been the most employed intervention across all disease groups, followed by decision assistance for health professionals [8] and delivery system design [9]. The delivery system design component encourages multidisciplinary teamwork among health professionals. Interventions that addressed delivery system design improved adherence to guidelines, patient service use, and physiological measures of disease [7]. Numerous scholars have advocated for manipulating the delivery system’s design to improve patient health care services [10,11,12,13]. 

However, there are limited studies about the integration of the management support and delivery system design components of CCM in diabetes management in the primary care setting in Pakistan.

This study is an attempt to address this gap by exploring the perceptions and opinions of patients with type 2 diabetes and healthcare professionals involved in the self-management of type 2 diabetes. The study focused on two important elements of CCM: patient self-management support (SMS) and delivery system design (DSD). More specifically, it assessed the suitability, practicality, and acceptability of these two core elements of CCM in improving the patients’ diabetes self-management approach, quality of life, risk behavior, knowledge and awareness of diabetes, and adherence to treatment.

## 2. Materials and Methods

### 2.1. Research Participants

Thirty patients with type 2 diabetes participated in this study. Patients with type 2 diabetes were recruited through purposive sampling from the outpatient clinics in rural medical centers in Pakistan. This small sample size was adequate as it was validated under “The Summary of an Urdu Version of Diabetes Self-Care Activities Measure” [14]. Additionally, Palinkas et al. [15] claimed that purposive sampling is most effective when data review and analysis occur concurrently with data collection. As a result, this sampling technique ensured the gathering, analysis, and evaluation of sufficient data. A modest sample size (*n* = 30 patients) allowed for an in-depth examination of individuals’ experiences with type 2 diabetes self-management. Lawton et al. [16] conducted a qualitative study of open-ended interviews with 32 individuals. As a result, this sample size is appropriate for the study’s objectives.

The participation of males and females was equal (50% each). The mean age of the participants was 52 years (range: 40–65 years) and the mean duration of time since diagnosis of Type 2 diabetes was 8 years (range: 2–13 years). The medical records accessed from the medical center of the hospital showed higher values of HbA1c 9% for both male and female patients (ranging between 2 and 13%). The mean value of the body mass index (BMI) was 29 Kg/m^2^.

The other group of participants consisted of 20 health-care professionals; there were 10 nurses and 10 general practitioners. The participation was equally represented by males and females. The details of the recruitment process, research teams, and qualitative analysis were discussed in [17].

The healthcare professionals and patients with type 2 diabetes involved were most concerned with the primary health-care approach to diabetes self-management. The following criteria were used to determine inclusion: (a) a minimum age of 45 years, (b) a diagnosis of type 2 diabetes five years’ prior, (c) agreed to openly communicate their experiences based on the interview guide, and (d) agreed to be audio-recorded. The inclusion of other healthcare professionals (aside from the doctors and nurses) such as dietitians, social workers, and pharmacists posed many difficulties in locating them in the rural areas and obtaining their consent to participate in the study. Therefore, they were not included in the study. All interviews were audio-recorded after obtaining participants’ consent. Semi-structured interview questions were guided by the self-management support and delivery system design of the CCM. Data collection continued until data saturation was reached.

### 2.2. Study Design

The aim of this study was to explore the perceptions and opinions of people with type 2 diabetes and healthcare professionals on the factors affecting the self-management of type 2 diabetes. The chronic care model was selected as the theoretical framework for this study and used to guide the qualitative thematic analysis based on the data collected during interviews. The reporting of the qualitative analysis complied with the consolidated criteria for reporting qualitative research (COREQ) guidelines (research teams and reflexivity, study design, analysis, and findings) [18]. In the literature, there was no evidence of a similar type of innovative approach in previous studies with the same idea and sample composition exploring different perspectives as described in this study.

**Figure 1:**
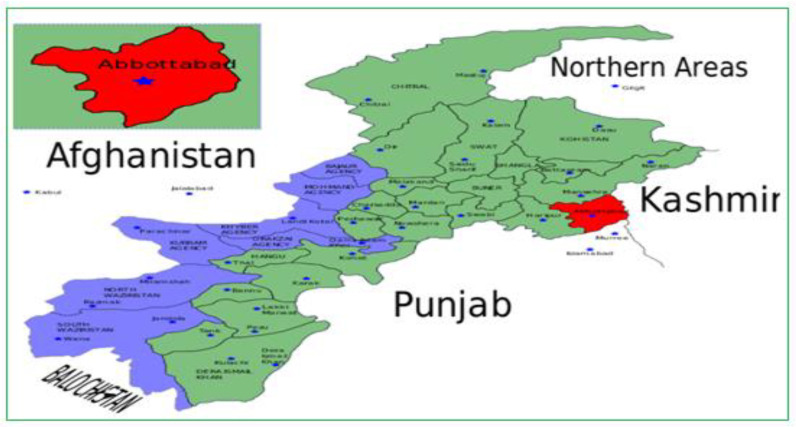
The study area (Abbottabad) shown on the map of Pakistan.

The study area, Abbottabad city is shown on the map of Pakistan in Figure 1.

The estimated population of Abbottabad city in 2010 was 1.1719 million [19]. The city is located in the north of Pakistan about 110 km from Islamabad (the capital city). Around 80% of the population live in rural areas, there are nineteen primary healthcare clinics in that area, and five of these clinics are associated with the hospital where this study was conducted [19,20].

### 2.3. Data Collection

The interviews with the 30 patients with type 2 diabetes and 20 healthcare professionals (10 general practitioners and 10 nurses) were conducted face-to-face in the Urdu language in a clinical setting at various medical centers of Al-Rehman hospital, Pakistan.

The main author took part in the semi-structured interviews along with an assisting nurse from the medical center. The main author moderated the interviews and prompted participants to share their experiences. The participants were encouraged to share their ideas on the questions asked. Each interview lasted 30–40 min. The semi-structured interviews were transcribed and translated from the Urdu language. Following an initial check for completion of data collection, all identifying information of the participants was removed and objective identifiers were used on the transcripts to ensure anonymity. The questionnaire used in this study was related to the elements of the chronic care model and the questions asked were the same for patients and healthcare professionals.

### 2.4. Data Analysis

The transcripts were imported into NVivo 11 Pro (QSR International, MA, USA, 2018) [21] and thematically analyzed [22,23]. The qualitative analysis of the semi-structured interviews was carried out using the chronic care model as a theoretical framework, and a methodological approach was carried out using a self-management framework proposed by Brewer-Lowry [24]. This approach allowed for a rich in depth understanding through a holistic framework and, therefore, was most suited to answer the research question in this study [25].

The research team addressed issues of rigor and trustworthiness based on a quality framework outlined by Meyrick [26]. Our research team was composed of three academic experts in diabetes and one local practice diabetes expert. The main researcher carried out the individual interviews but all other researchers and the local diabetes expert checked and discussed the collected data. The transcripts had all identifying information removed so that we were able to maintain confidentiality and anonymity during analysis.

## 3. Results

The statements made by the participants during the interviews were arranged into the subthemes related to the components of the chronic care model. The data analysis of 30 patients of diabetes and 20 healthcare professionals resulted in 340 statements from all the participants (*n* = 50) which were organized into six main themes of the chronic care model. As shown in Table 1, male and female patients with Type 2 diabetes made a total of 226 statements. The healthcare professionals made 114 statements. The “community resources” element or factor of chronic care model was most frequently mentioned (80 statements, 23%).

The element “healthcare system” was the least discussed factor (33 statements, 10%). Table 1 shows the various statements related to the CCM themes such as “delivery system”, self-management”, decision support”, and “clinical information”. All these elements of chronic care model fall under the practice level. The elements “community resources” and “health care system” fall under the community and system level.

**Figure 2:**
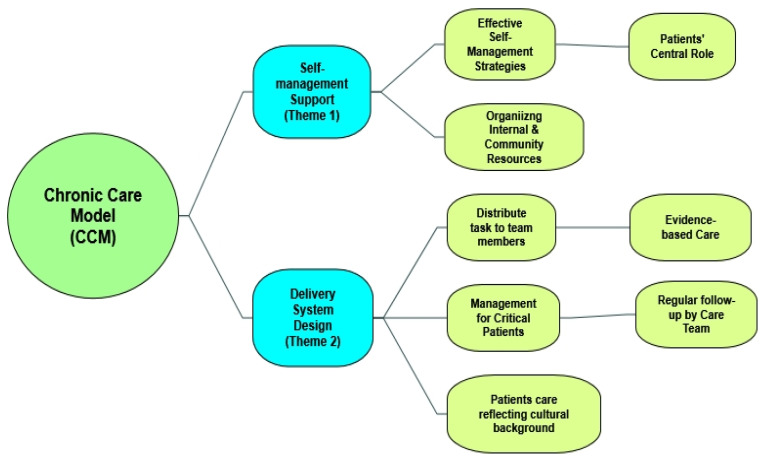
Subthemes of the two main themes of the chronic care model at the practice level.

From the main themes of CCM at practice level (Table 1), we discussed self-management support (SMS) and its subthemes (Table 2) and delivery system design (DSD) and its subthemes (Table 3) for the purpose of this study. Figure 2 provides the subthemes of the two main themes of the chronic care model at the practice level.

The interview discussions on the self-management of type 2 diabetes revealed three sub-themes and 68 statements highlighting the importance of self-management shown in (Table 2).

### 3.1. Subtheme 1: Patients’ Central Role in Managing Type 2 Diabetes

Table 2 shows that patients with type 2 diabetes were concerned with “patients’ central role in managing type 2 diabetes” (34 statements). Some patients stated that they had to manage their diabetes on their own with little help from family or health services.

One of the patients stated:

*…”It depends entirely on us how we manage our diabetes, there is no other help for me …my other friends with diabetes have the same problems”.*

(Patient_15).

Patients did not feel any challenges associated with impaired glycemic control, and hence did not view themselves as actively managing their illness—perhaps lack of knowledge about diabetes.

*…”It is not a great challenge for me to carry out self-management activities… in a family set-up, I don’t have any other option but to follow what others do”.*

(Patient _22).

There was a lack of realization by the patients that self-management is their own responsibility in managing diabetes; health professionals can only provide some guidelines on how to manage these activities.

One of the GPs explained:

*… “I think that from a patients’ perspective, there is no conscious self-management, it is just dealing with diabetes in daily life, patients do not consider diabetes a serious threat to their day to day life”.*

(GP_9).

Another GP mentioned:

*…”It is important for the patients to take care of their health and wellbeing by managing their diabetes”.*

(GP_10).

Nurses, in collaboration with general practitioners, played an important role by encouraging patients to set goals, identify barriers and challenges to self-management of type 2 diabetes, and monitor their own conditions.

One of the nurses stated:

*…”Patients play an important role in managing their diabetes…they have been told and explained what is required from them to remain healthy and avoid diabetes related complication”.*

(Nurse_5).

### 3.2. Subtheme 2: Effective Self-Management Support Strategies

Table 2 shows that patients with type 2 diabetes were also concerned with “self-management support strategies” (27 statements). Patients with diabetes identified very specific aspects that contributed to their feeling supported. For instance, when it came to exercise, patients felt that they were supported by their children. However, patients were unable to articulate specific reasons for the lack of support from health services.

*…”It is a great feeling that my children support and encourage me to go on walk and do some light exercises and sometime accompany me”.*

(Patient _25).

Many noted a lack of motivational assistance when it came to exercise. In general, patients felt supported in some aspects of self-management but primarily felt as if they were on their own to learn everything about living with diabetes.

*…”It is good that our health professionals provide medical advice but don’t spend much time to explain how to manage diabetes on a daily basis”.*

(Patient _28).

Some patients expressed a desire for educational/information classes in the local language to know more about diabetes and how to manage it on a day to day basis.

*…”It will be very helpful if we know in details about diabetes and its complications and the guidelines on how to manage it in a formal class room set up in a local language”.*

(Patient _30).

### 3.3. Subtheme 3: Organizing Internal and Community Resources

It was observed during the discussions and shown in Table 2 that healthcare professionals were unable to provide any statements on “the need to organize internal and community resources to support self-management outcomes”. This subtheme was only discussed by patients with type 2 diabetes (Total seven statements: three statements from male patients and four statements from female patients).

The other core element of the chronic care model “delivery system design” was an important factor that affected the self-management activities of the middle-aged population of the rural area. The aim of this core element of CCM is to facilitate routine proactive scheduled visits with general practitioners that incorporate patient goals, assisting individuals in maintaining optimal health, and enabling health systems to manage their resources more effectively. Table 3 shows that “distributing tasks among team members” (24 statements) and “interactions to support evidence-based care” (13 statements) were the most common concerns of participants with type 2 diabetes and health professionals.

Patients expressed that a teamwork approach is a better choice as it will optimize the general practitioner’s time and more patients will have access to the physicians.

*… “It will be helpful if some physicians’ duties are delegated to nurses and, after reviewing the nurses report, the physician could visit more patients during his/her allocated time for consultations”.*

(Patient_10)

The healthcare professionals found that case management services could help enhance the self-management activities outcomes. However, healthcare professionals perceived “case management for critical patients” as a way to improve the relationship with the “acute care organization”, instead of providing direct support to type 2 diabetes patients’ needs, such as self-management education or awareness.

The other interesting observation was on the statement “patient care reflecting their cultural background”, that is related to the need to provide care for patients with type 2 diabetes. That was important for male and female patients with type 2 diabetes but not for healthcare professionals as only one GP expressed this opinion.

All the patients under the sub-themes “patient care reflecting their cultural background” were of the opinion that self-management perceptions and care should reflect the “cultural background” of the people living in various rural areas of Pakistan.

Some patients believe that whatever their spiritual leader suggests or approves in relation to diabetes self-management will be a better option and acceptable to them.

One of the patients mentioned:

*“I want to consult my spiritual healer to see his opinion how to manage diabetes as some of my friends are feeling better after visiting him”.*

(Patient_12).

One of the GPs expressed:

*“My patient decided that self-management activities to be performed as per the direction of his spiritual healer including the treatment…... I believe my patient’s choice of treatment poses a risk to his well-being”.*

(GP_8).

## 4. Discussion

The literature suggests that the successful implementation of the CCM provides health professionals an effective framework for supporting diabetes self-management [27]. In line with previous studies [28,29,30,31], our findings showed that the self-management and delivery system design components of CCM are promising in diabetes management in the context of primary healthcare of Pakistan.

We have considered two key components of the CCM in this study to assess their relevance, feasibility, and acceptability in the primary healthcare system of Pakistan.

The patients’ central role in managing type 2 diabetes was found to be an important subtheme of the core element of self-management of the CCM. However, patients did not realize that self-management is their own responsibility in managing diabetes, while health professionals can only provide them some guidelines on how to manage these activities. This finding has significant implication for diabetes education and management as Fisher et al. [28] suggested that “quality clinical care and self-management are compatible and dependent on each other and without sound care, patient’s efforts may be misdirected and expert clinical care will fall far short of its potential, through patient failure to use prescribed medications to control his/her blood sugar or to implement its management plans” [28].

On the aspect of self-management support strategies, patients expressed their desire to have educational/information classes in the local language, which suggests that diabetes education should be delivered in the local language.

The healthcare professionals found that case management services could help enhance self-management activities’ outcomes. However, healthcare professionals perceived “case management for critical patients” as a way to improve the relationship with the “acute care organization”, instead of providing direct support to type 2 diabetes patients’ needs, such as self-management education or awareness.

The other interesting observation was that “patients are influenced by their cultural background”. In line with our finding, other studies found that patient understanding and beliefs about health and illness might be shaped by historical and local contexts [32]. 

Hassan et el. [33] found that self-management interventions conducted in community settings and delivered by peer coaches living in the same community were more likely to lead to significant improvement in type 2 diabetes self-management and clinical outcomes.

The two subthemes of delivery system design: distributing tasks among team members and planned interactions to support evidence-based care were the most commonly mentioned subthemes among the participants and health professionals. The teamwork approach was considered the most helpful in optimizing the general practitioners’ time. This might be related to the shortage of general practitioners in rural areas of Pakistan and therefore, more patients would have access to the physicians with a teamwork approach [34,35].

### 4.1. Strengths and Limitations

The strength of this study was to use the two key components of the chronic care model to assess their relevance, feasibility, and acceptability in the primary healthcare system of rural areas of Pakistan. The study highlighted the role of the healthcare system in diabetes self-management outcomes. The previous studies did not address this issue and only highlighted patient-related factors.

This study recruited participants from two clinics affiliated with Abbottabad’s major hospital. As such, the study’s findings may not accurately reflect the self-management requirements of all diabetes patients in Pakistan. Future research should include patients from a variety of settings in rural areas to ensure the generalizability of the findings. Second, the authors gathered data on the experiences of selected patients with type 2 diabetes self-management at a specific point in time; hence, the results may not truly reflect individuals’ perspectives over time.

### 4.2. Relevance to Clinical Practice

The findings of this study emphasize several crucial areas and addressing these areas may result in an improvement in the self-management outcomes of patients with type 2 diabetes. The two main themes of CCM at practice level namely the self-management support (SMS) and delivery system design (DSD) were discussed in detail with the patients of type 2 diabetes. Participants expressed their need for self-management education suggesting a need for diabetes education and information in rural areas.

## 5. Conclusions

The chronic care model provides a solid foundation on which to promote self-management, and the model provided an effective framework for supporting diabetes self-management education and support. This study explored the perceptions and opinions of patients with type 2 diabetes and healthcare professionals on the various subthemes of the two core elements of chronic care model, affecting the self-management of type 2 diabetes and provided evidence that the elements that most frequently impact health and functional status and quality of life were self-management support and delivery system design, particularly when used in combination.

These two elements of the chronic care model are most suitable and feasible for implementation in future to improve the overall efficiency of the health care system of rural area of Pakistan. That implementation will help the middle-aged population of Pakistan to have awareness about diabetes and its complications and properly manage their diabetes. As a result, building systems that include accessible, long-term diabetes self-management education services that influence health outcomes has wide-ranging public health implications.

## Figures and Tables

**Table 1:** Number of factors affecting the self-management of type 2 diabetes outcome.

Themes of the Model (CCM)	Type 2 Diabetes Patients		Health Professionals	Total
Practice Level	*Male*	*Female*		*N* (%)
Delivery System (DSD)	20	10	30	60 (18)
Self-management (SMS)	22	25	21	68 (20)
Decision Support	19	10	20	49 (14)
Clinical Information	20	19	11	50 (15)
Community and System Level				
Community Resources	20	40	20	80 (23)
Health Care System	10	11	12	33 (10)
Total Statements	111	115	114	340 (100)

**Table 2:** Self-management of Type 2 diabetes outcome.

Subthemes	Diabetes Patients		Health Professionals	Total
	M	F		
Patients’ central role in managing Type 2 diabetes	11	12	11	34
Use of effective self-management support strategies	8	9	10	27
Organizing internal and community resources	3	4	0	07
**Total Statements**	**22**	**25**	**21**	**68**

**Table 3:** Delivery system design outcome.

Subthemes	Diabetes Patients		Health Professionals	Total
	M	F		
Distribute tasks among team members	9	4	11	24
Interactions to support evidence-based care	3	0	10	13
Provide clinical case management for critical patients	1	0	5	06
Ensure regular followup by the care team	4	1	4	09
Patient care reflecting their cultural background	3	4	1	08
**Total Statements**	**20**	**9**	**30**	**60**

## Data Availability

The data used to generate the results in the paper are not available to share.
